# Medical College Notes

**Published:** 1891-11

**Authors:** 


### Medical College Notes.

The College of Physicians and Surgeons, of Chicago, Ill., announces a non-resident course of instruction in the following terms:

1. Non-residents may matriculate to take the first year's course in the same manner and under the same conditions as if they proposed to take a resident course. (*V.* Catalogue).

2. Non-resident students will be required to select a preceptor satisfactory to the secretary, and one who is willing to coöperate with the faculty in conducting the year's work, and give his certificate for the same at the end of the year.

3. Non-resident students must do the prescribed work and make satisfactory weekly reports of progress in the manner provided by the faculty.

4. The course covers thirty weeks, and not more than five weekly reports may prove unsatisfactory without debarring the student from the credit of the course.

5. When a student can furnish evidence of having already taken the work in the prescribed non-resident course, he will be assigned an equivalent from a special course.

6. Students who have taken the non-resident course in a satisfactory manner, and have shown by the weekly examinations that they have done the work thoroughly and intelligently, will receive certificates from the secretary, which, with the certificate of their preceptor, will be taken at this college in lieu of one year's study on a four years' course. Address,

DR. BAYARD HOLMES, *Secretary*.

240 Washington Ave., Chicago, Ill.

